# High-Flow Nasal Cannula for Peri-Intubation Oxygenation in Pediatric Rapid Sequence Intubation: A Narrative Best-Evidence Review

**DOI:** 10.7759/cureus.103454

**Published:** 2026-02-12

**Authors:** Christopher J Walker, Brianna Yanover, Stephanie Bijos

**Affiliations:** 1 Osteopathic Medicine, Nova Southeastern University Dr. Kiran C. Patel College of Osteopathic Medicine, Clearwater, USA; 2 Pediatrics, Broward Health Medical Center, Fort Lauderdale, USA

**Keywords:** apneic oxygenation, high-flow nasal cannula, hypoxemia prevention, neonatal airway management, pediatric intubation, rapid sequence intubation

## Abstract

Pediatric rapid sequence intubation (RSI) carries a high risk of peri-intubation hypoxemia because children have limited oxygen reserve and desaturate rapidly during apnea. High flow nasal cannula (HFNC) is an appealing adjunct because it can deliver high, humidified flows that reduce room air entrainment and support a more stable upper airway oxygen concentration, with modest flow and leak-dependent distending pressure and upper airway dead space washout. Across pediatric perioperative and sedation studies and in acute care cohorts, the most consistent signal is improved oxygenation reliability, with fewer or less severe desaturation events and fewer rescue interventions, rather than a predictable extension of safe apnea time, which remains variable across settings. Adult peri-intubation trials provide a comparator context and reinforce that the observed benefit depends on baseline hypoxemia and the comparator strategy, often favoring NIV in severe hypoxemia, limiting direct generalization to children. Pediatric RSI-specific randomized trials are needed with standardized flow protocols and clinically meaningful endpoints, including severe desaturation rates, nadir SpO₂, time spent below clinically relevant thresholds, need for rescue ventilation or interrupted laryngoscopy, first pass success, and adverse events.

## Introduction and background

Rapid sequence intubation (RSI) in children carries a disproportionate risk of peri-intubation hypoxemia because pediatric patients have limited oxygen reserve and deteriorate rapidly during apnea [1]. Compared with adults, children have a smaller functional residual capacity (FRC) and higher oxygen consumption, which shortens the time to clinically significant desaturation once ventilation ceases [1]. These physiologic constraints are compounded by developmental airway anatomy and procedural factors that can prolong laryngoscopy and increase the likelihood of interrupted attempts, making hypoxemia a central driver of morbidity during pediatric airway management [2,3]. Observational pediatric airway data show that desaturation events are common during emergency airway management and are associated with younger age and adverse procedural characteristics such as prolonged attempts and esophageal intubation, underscoring the need for strategies that maintain oxygenation throughout induction and airway manipulation [4-7]. Physiologic studies similarly illustrate how rapidly oxygen saturation can decline in infants and young children compared with adults, highlighting that pediatric RSI often occurs on a narrow margin of safety where oxygenation support is critical [4,8].

RSI refers to the rapid administration of an induction agent and a neuromuscular blocker to facilitate tracheal intubation during a planned apneic interval while minimizing aspiration risk. In this setting, apneic oxygenation describes oxygen delivery during the apneic period between induction and paralysis and the restoration of ventilation. “Safe apnea time” is commonly used to describe the practical duration of apnea before oxygen desaturation reaches a clinically important threshold. In practical terms, a conventional low-flow nasal cannula delivers oxygen at flow rates that may be substantially lower than peak inspiratory demand, whereas a high-flow nasal cannula (HFNC) delivers warmed, humidified oxygen at higher flow rates intended to better maintain the upper-airway oxygen concentration. Clinically, peri-intubation hypoxemia may interrupt laryngoscopy and prompt rescue ventilation.

Apneic oxygenation is widely used during peri-intubation care in an effort to blunt desaturation during apnea [9,10]. In pediatrics, however, the practical effectiveness of conventional low-flow nasal cannula appears limited in the settings that matter most (e.g., prolonged apnea, high inspiratory demand, and physiologic fragility), where desaturation can occur despite its use [11]. This limitation is clinically relevant because pediatric RSI is frequently performed in children with minimal reserve, and even brief delays due to positioning, suctioning, equipment adjustments, or interrupted attempts can meaningfully increase hypoxemia risk. Accordingly, the key question is not whether apneic oxygenation is used, but whether the selected strategy can maintain oxygenation reliably despite real-world procedural interruptions.

HFNC has emerged as a potential strategy to address these vulnerabilities because it delivers heated, humidified oxygen at flow rates that meet or exceed inspiratory demand and may maintain more stable upper-airway oxygenation than low-flow approaches [12-14]. HFNC is therefore often framed not as a guarantee of longer “safe apnea time,” but as a means of improving oxygenation reliability (e.g., fewer desaturation events or rescue interventions), which may be more clinically meaningful during pediatric RSI [11,15].

Direct randomized trials evaluating HFNC specifically during pediatric RSI remain limited; thus, current practice often relies on indirect evidence from perioperative and sedation studies, ED/PICU cohorts, neonatal/infant respiratory support data, and adult peri-intubation trials [16-25]. Adult evidence provides useful comparator context but suggests that benefit is influenced by baseline hypoxemia and the comparator used, and therefore cannot be directly generalized to children [9-11,26]. Accordingly, this narrative best-evidence review synthesizes physiologic rationale and the best available pediatric and comparator evidence relevant to HFNC during RSI, clarifies where HFNC is most likely to improve peri-intubation oxygenation reliability, and outlines research priorities to address key evidence gaps in pediatric RSI [3,4,6,9-11,18-20,23-25,27-30].

## Review

Methods

Review Design and Scope

We conducted a best-evidence narrative review to synthesize the physiologic rationale and clinical outcomes for HFNC used for peri-intubation oxygenation in children, with a primary focus on RSI and airway procedures in acute care settings. Because pediatric RSI-specific HFNC trials are limited, we also included closely related pediatric contexts that inform mechanism and feasibility (e.g., procedural sedation, anesthesia induction, bronchiolitis/respiratory distress care, and neonatal airway practice). Adult peri-intubation trials and network meta-analyses were included to contextualize how baseline hypoxemia and comparator selection (e.g., facemask vs NIV/CPAP) influence observed effectiveness, while recognizing that these data cannot be directly extrapolated as proof of pediatric benefit. Because this was a narrative best-evidence review with qualitative synthesis rather than a PRISMA (Preferred Reporting Items for Systematic reviews and Meta-Analyses)-guided systematic review, we did not perform a PRISMA flow diagram or formal risk-of-bias appraisal.

Data Sources and Search Strategy

We identified candidate studies between September 2025 and January 15, 2026, using an iterative, best-evidence approach. Searches were performed in Scopus and Web of Science Core Collection to maximize cross-disciplinary capture and citation-linking functionality. We also used Elicit as a literature discovery tool to surface potentially relevant peer-reviewed articles indexed across major bibliographic sources; all Elicit-surfaced citations were manually verified by accessing the original journal record (publisher page and/or database entry) to confirm bibliographic details, relevance, and study context. We then performed backward and forward citation screening from key systematic reviews and primary studies to identify additional eligible papers.

Search concepts combined HFNC terminology with peri-intubation/airway management concepts and pediatric populations/settings. Keywords included: “high-flow nasal cannula,” “high-flow nasal oxygen,” “HFNC,” “HFNO,” “heated humidified high flow,” combined with “rapid sequence intubation,” “RSI,” “tracheal intubation,” “intubation,” “preoxygenation,” “apneic oxygenation,” “laryngoscopy,” “airway management,” and population/setting terms including “pediatric,” “paediatric,” “infant,” “child,” “neonate,” “emergency department,” “ED,” “PICU,” “ICU,” “procedural sedation,” and “anesthesia.”

Because this was a best-evidence narrative review, the search was designed to identify the most relevant evidence rather than to be exhaustive; we did not use a single fixed Boolean strategy with record counts, and additional eligible studies may exist outside the included set.

Eligibility Criteria

The review included English-language studies meeting one or more of the following criteria: (1) pediatric peri-intubation airway management studies evaluating HFNC as preoxygenation and/or apneic oxygenation during intubation or airway procedures; (2) pediatric procedural sedation or anesthesia induction studies evaluating HFNC for prevention of hypoxemia, included as operationally relevant surrogates; (3) PICU and ED pediatric respiratory distress trials, cohorts, and meta-analyses reporting oxygenation outcomes and escalation or rescue interventions to inform feasibility and real-world performance; (4) neonatal studies relevant to intubation practice or HFNC physiology to contextualize age-specific respiratory physiology and airway risk; and (5) adult peri-intubation/preoxygenation systematic reviews, network meta-analyses, and trials included for design and comparator context and mechanistic interpretation, not as direct evidence of pediatric efficacy. The review excluded studies focused solely on post-extubation support without peri-intubation oxygenation endpoints, studies in which HFNC was not a primary exposure or comparator relevant to peri-intubation oxygenation, and reports without extractable airway-relevant outcomes.

Study Selection and Data Extraction

Titles and abstracts were screened for relevance to HFNC in pediatric peri-intubation care or closely related pediatric airway contexts (Table 1). Full texts were assessed for eligibility. For included studies, we extracted: setting (ED/PICU/OR/ward), age group and acuity, HFNC protocol (flow strategy and timing as preoxygenation vs apneic oxygenation), comparator (standard oxygen devices vs NIV/CPAP), and airway context (RSI, laryngoscopy, procedural sedation, induction). Outcomes prioritized clinically meaningful oxygenation measures during airway management: incidence of desaturation/hypoxemia events, minimum SpO₂, need for rescue oxygenation/ventilation interventions, and intubation attempt characteristics when available. CO₂ endpoints (EtCO₂/PaCO₂) and surrogate measures of apnea time were extracted when reported. Baseline oxygenation severity (e.g., pre-procedure SpO₂ or acute respiratory failure status) was recorded to support interpretation by risk strata. Final study inclusion was determined by consensus among the authors based on relevance to pediatric peri-intubation oxygenation with HFNC or closely related surrogate contexts.

**Table 1:** Key characteristics and findings of included studies on HFNC and peri-intubation oxygenation AE(s): adverse event(s); ED: emergency department; PICU: pediatric intensive care unit; ICU: intensive care unit; RSI: rapid sequence intubation; HFNC: high-flow nasal cannula; HFNO: high-flow nasal oxygen; NIV: noninvasive ventilation; CPAP: continuous positive airway pressure; O₂: oxygen; FiO₂: fraction of inspired oxygen; SpO₂: peripheral oxygen saturation; PaO₂: arterial oxygen partial pressure; PEEP: positive end-expiratory pressure; CO₂: carbon dioxide; WOB: work of breathing; EELV: end-expiratory lung volume; FRC: functional residual capacity; VO₂: oxygen consumption; RCT: randomized controlled trial; MA: meta-analysis; NMA: network meta-analysis; nCPAP: nasal continuous positive airway pressure; EIT: electrical impedance tomography

Citation	Evidence tier	Population/setting	Intervention/focus	Key findings
Trachsel et al., 2022 [1]	Mechanistic/physiology	Pediatrics (general)	Respiratory physiology; oxygen reserve/FRC/VO₂	Low FRC and high VO₂ lead to rapid oxygen desaturation during apnea
Hsu et al., 2021 [2]	Mechanistic/physiology	Pediatrics (general)	Airway management principles/complications	Hypoxemia is common during pediatric airway management, so preparation and rescue planning are essential
Stein et al., 2024 [3]	Direct peri-intubation (peds/neonatal)	Pediatrics; difficult airway registry	Airway management outcomes; time trends	Airway difficulty and technique factors are associated with higher AE rates
Rinderknecht et al., 2015 [4]	Direct peri-intubation (peds/neonatal)	Pediatrics; ED (RSI/intubation)	Factors associated with oxyhemoglobin desaturation	Younger age and longer attempts are associated with more frequent oxygen desaturation
Lee et al., 2016 [5]	Direct peri-intubation (peds/neonatal)	Pediatrics; multicenter intubations	Attempts vs outcomes/complications	A higher number of attempts is associated with increased complications and hypoxemia
Li et al., 2018 [6]	Direct peri-intubation (peds/neonatal)	Pediatrics; PICU (intubation)	Desaturation frequency and hemodynamic AEs	Oxygen desaturation is common and is associated with hemodynamic AE(s)
Foglia et al., 2019 [7]	Direct peri-intubation (peds/neonatal)	Neonates (registry/practice)	Practice patterns and outcomes	Practice varies widely, and more attempts are associated with more AE(s)
Else and Kovatsis, 2020 [8]	Mechanistic/physiology	Pediatrics (general)	Oxygenation strategies during airway procedures	HFNC may improve oxygenation reliability more consistently than extending apnea time
Lyons and Callaghan, 2019 [9]	Mechanistic/physiology	General physiology (apneic oxygenation)	Apneic oxygenation mechanisms	Apneic oxygenation can support oxygen delivery during apnea, but does not remove CO₂
Fuchs et al., 2024 [10]	Direct peri-intubation (peds/neonatal)	Pediatrics; intubation (varied settings)	Apneic oxygenation (various devices)	Apneic oxygenation may reduce hypoxemia, but results are highly heterogeneous
Zhao et al., 2025 [11]	Indirect pediatric surrogate	Pediatrics; perioperative	HFNC perioperative hypoxemia outcomes	HFNC reduces hypoxemia and rescue interventions compared with standard oxygen
Spoletini et al., 2015 [12]	Comparator adult	Adults (general)	HFNC/HFNO mechanisms and clinical use	HFNC can provide more stable FiO₂, reduce upper airway dead-space rebreathing, and generate modest PEEP, with stronger effects at higher flows
Kwon, 2020 [13]	Indirect pediatric surrogate	Pediatrics (general)	HFNC indications and outcomes	HFNC is generally feasible and often improves oxygenation and WOB
Nishimura, 2019 [14]	Mechanistic/physiology	General (device/physiology)	Device characteristics, flows, and interfaces	HFNC performance depends on flow settings and interface fit/leak
Kuitunen et al., 2024 [15]	Indirect pediatric surrogate	Pediatrics (general)	HFNC for non-bronchiolitis indications	Pediatric RCT evidence is limited, with mixed effects on hypoxemia
Nolasco et al., 2022 [16]	Mechanistic/physiology	Pediatrics (general)	Mechanisms + applications	Clinical benefit depends on flow rate, leak, and technique
Asseri et al., 2021 [17]	Indirect pediatric surrogate	Pediatrics; PICU	Indications, safety, outcomes	HFNC is generally safe and may reduce escalation in some patients
Jhou et al., 2020 [18]	Comparator adult	Adults; ICU (intubation)	HFNC apneic oxygenation vs comparators	HFNC performs similarly to standard oxygen overall, with possible benefit in selected subgroups
Kuo et al., 2022 [19]	Comparator adult	Adults; perioperative anesthesia	Preoxygenation technique outcomes	HFNC may increase PaO₂ and apnea time vs standard oxygen, but effects on hypoxemia are mixed
Lee et al., 2025 [20]	Indirect pediatric surrogate	Pediatrics; procedural sedation	Oxygen supplementation strategies	Oxygen delivery strategy during sedation changes hypoxemia rates
Alsabri et al., 2025 [21]	Indirect pediatric surrogate	Pediatrics; acute care (ED/PICU/ward)	HFNC vs standard oxygen (resp distress)	Early HFNC improves respiratory distress and may reduce treatment failure/escalation
Zifeng et al., 2025 [22]	Indirect pediatric surrogate	Perioperative procedural population	Post-op atelectasis outcomes	HFNC improved oxygenation and reduced postoperative atelectasis
Song et al., 2022 [23]	Comparator adult	Adults; perioperative anesthesia	Pre- and apneic oxygenation	HFNC may improve PaO₂/apnea time during induction, with inconsistent effects on hypoxemia
Coletti et al., 2017 [24]	Indirect pediatric surrogate	Pediatrics; PICU	Utilization patterns, outcomes	HFNC is widely used and feasible in the PICU, but protocols vary
Luo et al., 2019 [25]	Indirect pediatric surrogate	Pediatric respiratory distress	HFNC vs standard oxygen/nCPAP	HFNC reduces failure vs standard oxygen and is comparable to CPAP in selected patients
Nielsen et al., 2018 [26]	Mechanistic/physiology	Infant/pediatric/adult models	Expiratory pressure, ventilation effects	HFNC generates modest PEEP that varies with flow, leak, and mouth position
Wang et al., 2025 [27]	Comparator adult	Adults; procedural sedation (obesity)	HFNC vs standard O₂; hypoxia incidence	HFNC reduced hypoxemia vs standard oxygen in high-risk obese patients
Pitre et al., 2025 [28]	Comparator adult	Adults; critically ill (preoxygenation)	Preoxygenation strategies include NIV, HFNC	NIV performs best overall; HFNC often outperforms facemask/standard oxygen in some comparisons
Tang et al., 2025 [29]	Comparator adult	Adults; emergency surgery (RSI)	HFNC vs comparators during RSI	HFNC may increase PaO₂/apnea time during RSI, with mixed effects on hypoxemia and nadir SpO₂
Zhong et al., 2024 [30]	Comparator adult	Adults; preoxygenation (mixed settings)	Preoxygenation methods	NIV/CPAP typically ranks highest; HFNC often outperforms standard oxygen
Liew et al., 2020 [31]	Mechanistic/physiology	Preterm infants	HFNC physiologic effects	HFNC effects on mechanics and oxygenation are flow-dependent in preterm infants
Nascimento et al., 2022 [32]	Mechanistic/physiology	Infants; bronchiolitis	Flow vs EELV (FRC surrogate)	Higher HFNC flow increased EELV, suggesting improved lung recruitment
Numa and Newth, 1996 [33]	Mechanistic/physiology	Pediatrics; infants/children	Dead space measurements	Children have proportionally larger dead space, supporting faster oxygen desaturation during apnea
Klotz et al., 2020 [34]	Indirect pediatric surrogate	Pediatrics; procedural sedation	HFNC vs standard care; hypoxemia outcomes	HFNC was feasible and suggested a possible reduction in hypoxemia (pilot data)
Mikalsen et al., 2016 [35]	Indirect pediatric surrogate	Pediatrics (general)	HFNC use; mechanisms/outcomes	Summarizes HFNC mechanisms/indications; outcomes vary by setting and protocol
Bressan et al., 2013 [36]	Indirect pediatric surrogate	Pediatrics; ward (bronchiolitis)	HFNC oxygenation/work of breathing	HFNC improved oxygenation/clinical distress; close monitoring for failure is needed
Nishimura, 2016 [37]	Comparator adult	Adults (general)	Physiology + clinical benefits	HFNC benefit depends on patient selection, setting, and flow settings
Waheed et al., 2025 [38]	Comparator adult	Adults; ED (intubation)	Low-flow vs HFNC apneic oxygenation	HFNC vs low-flow apneic oxygenation showed modest, protocol-dependent differences
Xu et al., 2018 [39]	Comparator adult	Adults; acute respiratory failure/post-extubation	HFNC vs comparators	HFNC improves comfort/oxygenation and may reduce reintubation in some settings

Quality Considerations and Synthesis

Given heterogeneous designs and the narrative best-evidence aim, we assessed methodological credibility qualitatively across key domains, including randomization/allocation procedures in trials, confounding and selection bias in observational studies, completeness of reporting, and appropriateness of the comparator strategy and HFNC protocol standardization. Evidence was synthesized thematically and reported descriptively; no quantitative pooling (meta-analysis or meta-regression) was performed. Findings were organized into: (1) pediatric physiologic rationale for desaturation vulnerability and HFNC mechanisms, (2) direct and surrogate pediatric peri-intubation evidence, (3) ED/PICU pediatric experience in respiratory distress and rescue/escalation outcomes, and (4) adult peri-intubation literature to highlight the impact of baseline hypoxemia and comparator strategy on apparent effectiveness. We did not perform a formal risk-of-bias tool-based appraisal; instead, we used this qualitative credibility assessment to guide interpretation of study findings.

Physiology of apneic oxygenation and high-flow therapy

HFNC delivers heated, humidified oxygen at flow rates that meet or exceed inspiratory demand and may maintain more stable upper airway oxygenation than low-flow approaches [12-14]. HFNC can also generate modest, flow-dependent distending pressure and may reduce upper airway dead space, effects that are influenced by leak, technique, and flow selection [16,26,31]. Consistent with this, HFNC-associated distending pressure may increase end-expiratory lung volume/FRC and help mitigate atelectasis, particularly in infants [32]. These considerations are particularly relevant in children, who have proportionally larger anatomic dead space and higher oxygen consumption than adults, contributing to rapid desaturation during apnea [1,33].

Review of the literature

Pediatric Evidence Relevant to HFNC During Intubation

Large observational and trial data identify hypoxemia as a leading peri-anesthetic complication in children and a key driver of morbidity, underscoring the value of strategies that stabilize oxygenation during airway manipulation [11]. In pediatric perioperative and procedural contexts, conventional low-flow nasal cannulas may be ineffective in high-risk situations, and oxygen desaturation can still occur, particularly when physiologic reserve is low or apnea is prolonged [15]. HFNC addresses these limitations by delivering heated, humidified oxygen at flow rates that meet or exceed inspiratory demand, which may better maintain upper airway oxygen concentration during induction and laryngoscopy [8,16]. Accordingly, weight-based protocols (e.g., ≥2 L/kg/min in infants and small children) are commonly used as a physiologically grounded approach to high-flow delivery during airway management [16].

Pediatric Surrogate Evidence (Perioperative, Procedural Sedation, ED/PICU Cohorts)

Across broader pediatric respiratory populations, evidence supports HFNC feasibility and safety in high-acuity children. ED and PICU cohort data demonstrate that HFNC can produce early improvements in physiologic distress (e.g., oxygenation, work of breathing) and can reduce escalation to CPAP or intubation in many settings [17,21,24]. In pediatric acute respiratory failure, HFNC has been associated with reduced treatment failure and/or intubation compared with standard oxygen and has demonstrated non-inferiority to CPAP across several pediatric cohorts [16,25]. In pediatric perioperative and procedural sedation studies (used here as operationally relevant surrogates rather than RSI-specific evidence), HFNC has been associated with fewer hypoxemia events and fewer rescue interventions than conventional oxygen, while effects on “safe apnea time” are inconsistent; at least one pediatric sedation trial showed no advantage in apnea-related stability despite adequate oxygenation [20,22,34]. Collectively, these findings suggest HFNC’s most consistent pediatric signal is fewer hypoxemia events and fewer rescue interventions, while effects on apnea duration are less consistent [11,15]. Real-world implementation and cohort studies in bronchiolitis, pneumonia, and mixed respiratory distress likewise report improved oxygenation and reduced work of breathing with generally low failure rates [13,17,25,35,36].

Adult Comparator Evidence Informing HFNC During Intubation

Although adult studies cannot be directly generalized to children, given important physiologic differences, they provide useful context for HFNC’s potential role during intubation. During ICU intubation, HFNC has been shown to improve the lowest SpO₂ and reduce severe hypoxemia compared with non-rebreather masks in mildly hypoxemic adults, with meta-analytic data suggesting non-inferior oxygenation outcomes versus standard care and context-dependent benefits [18,28,37]. In peri-intubation settings outside the ICU, meta-analyses of rapid sequence induction in emergency surgery report higher post-intubation PaO₂ and longer apnea time with HFNC compared with facemask oxygenation, although effects on desaturation incidence and nadir SpO₂ are less consistent [19,23,29]. Network meta-analyses of preoxygenation in critically ill adults generally identify noninvasive ventilation as most protective against hypoxemia, while still suggesting that HFNC can reduce hypoxemia compared with facemask oxygenation and prolong safe apnea time with a similar or favorable complication profile [28,30]. Outside the intubation setting, adult procedural sedation trials, including those in high-risk obesity cohorts, report reduced hypoxemia with HFNC compared with standard oxygen, supporting feasibility but not providing RSI-specific evidence [27].

Mechanistically, HFNC’s ability to maintain a stable high FiO₂, generate low-level distending pressure, and wash out anatomic dead space is well described in adults and becomes more flow-dependent at higher settings [37-39]. Adult data therefore support describing HFNC as a safe, non-inferior, and sometimes superior adjunct for preoxygenation and apneic oxygenation during intubation, with benefits modulated by baseline hypoxemia, setting, and comparator.

Practical Implications for Pediatric RSI

Based on the physiologic rationale and indirect pediatric evidence, HFNC is best considered an adjunct to optimized preoxygenation and apneic oxygenation rather than a replacement for established rescue strategies [11,16]. HFNC may be most useful in infants and young children and in patients with bronchiolitis, pneumonia, or mixed respiratory distress who are prone to rapid desaturation [1,25,36]. Flow should be selected to meet or exceed inspiratory demand, and weight-based strategies (commonly ≥2 L/kg/min in smaller children, transitioning to fixed higher flows with increasing size) align with the principle that benefit is flow-dependent and technique-dependent [16,35]. HFNC can be initiated during preoxygenation and continued through induction and laryngoscopy; however, clinicians should anticipate flow- and leak-dependent variability in distending pressure and should maintain readiness to escalate oxygenation support, including NIV/CPAP in appropriate patients, if oxygenation deteriorates [26,28,30].

Future Research Priorities and Recommended Trial Endpoints

The evidence base remains heterogeneous and largely indirect, spanning perioperative and sedation trials, ED and PICU cohorts, neonatal/infant respiratory support data, and adult peri-intubation trials, with variability in flow strategies, comparators, baseline hypoxemia, and outcome definitions [16-25]. Pediatric RSI trials should therefore prioritize standardized flow protocols and clinically relevant comparators (e.g., optimized facemask/non-rebreather strategies, and NIV/CPAP where appropriate for more severe hypoxemia) given the comparator-dependent effects seen in adults [28,30]. Outcomes should emphasize oxygenation reliability and procedural continuity rather than apnea time alone, including severe desaturation rates, nadir SpO₂, time below saturation thresholds, interruptions for rescue ventilation, escalation to alternative oxygenation strategies, first-pass success, time to intubation, and complications such as aspiration or hemodynamic instability [4,6,11]. Trainee/operator-level performance outcomes are rarely reported in the existing pediatric surrogate literature, so future pediatric RSI trials should explicitly capture and report first-pass success and other procedural metrics stratified by operator experience (e.g., trainee vs attending). Key design considerations and recommended endpoints are summarized in Table 2.

**Table 2:** Future research priorities and recommended trial endpoints for pediatric RSI studies using HFNC RSI: rapid sequence intubation; HFNC: high-flow nasal cannula; NIV: noninvasive ventilation; CPAP: continuous positive airway pressure; SpO₂: peripheral oxygen saturation; FiO₂: fraction of inspired oxygen; EtCO₂: end-tidal carbon dioxide; PaCO₂: arterial partial pressure of carbon dioxide; ED: emergency department; PICU: pediatric intensive care unit

Domain	What future pediatric RSI trials should specify	Why it matters
Study population	Specify setting (ED/PICU/OR), airway indication (RSI vs other peri-intubation airway management), and inclusion criteria (e.g., respiratory distress phenotypes)	Reduces “apples-to-oranges” comparisons and improves generalizability
Stratification	Pre-specify strata by age/weight and baseline oxygenation severity (e.g., SpO₂/FiO₂ category; presence of acute respiratory failure)	Effect size is likely different in infants vs older children and in mild vs severe hypoxemia
HFNC protocol	Standardize flow strategy (e.g., weight-based approach), timing (preoxygenation only vs continued through laryngoscopy), and FiO₂ targets	HFNC performance is flow- and technique-dependent; inconsistent protocols drive heterogeneity
Technique factors	Report interface sizing/fit, leak mitigation strategies, and concurrent airway maneuvers during preoxygenation; document mouth position (open/closed) when feasible	These factors materially change the delivered FiO₂ and distending pressure
Comparator arms	Use clinically relevant, explicitly optimized comparators (e.g., optimized facemask/nonrebreather preoxygenation), and include an NIV/CPAP arm when feasible for moderate-to-severe hypoxemia	Adult data show outcomes are highly comparator-dependent; weak comparators inflate apparent benefit
Co-interventions	Standardize or report induction agents, paralysis strategy, positioning, suctioning, and apneic oxygenation use in comparator groups	Minimizes confounding from airway bundle variability
Primary outcome (recommended)	Severe desaturation incidence (pre-specify a threshold such as SpO₂ <80% or <85%)	Clinically meaningful, patient-safety–relevant, and less “surrogate” than apnea time
Key oxygenation outcomes	Nadir SpO₂; time below a pre-specified SpO₂ threshold; hypoxemia event rate; need for rescue oxygenation/ventilation	Captures “oxygenation reliability,” which appears to be the most consistent signal across pediatric surrogate data
Procedural performance outcomes	Interrupted laryngoscopy/need for rescue ventilation; number of attempts; first-pass success; attempt duration/time to intubation; use of adjuncts; and, when feasible, stratify key outcomes by operator experience (e.g., trainee vs attending)	Links oxygenation strategy to real-world airway performance and procedural continuity
Ventilation/CO₂ outcomes	EtCO₂ or PaCO₂ (when available); hypercapnia events; post-intubation ventilation parameters	Addresses physiologic tradeoffs and helps interpret “apneic oxygenation” claims
Safety outcomes	Aspiration/regurgitation; hypotension/bradycardia; cardiac arrest; barotrauma; device intolerance	Ensures benefits are not offset by harms
Reporting standards	Provide clear definitions of RSI and apneic oxygenation, standardized outcome definitions, and complete HFNC/comparator protocol details	Improves reproducibility and reduces citation-to-claim drift

Discussion

The central implication of the current literature is that HFNC, when considered for pediatric RSI, should be framed primarily as an oxygenation reliability tool rather than a guaranteed way to extend “safe apnea time.” Across perioperative and sedation-focused pediatric trials and syntheses, HFNC is most consistently associated with fewer desaturation events, higher minimum SpO₂, and fewer rescue airway interventions compared with conventional oxygen strategies, while effects on apnea duration and CO₂ endpoints are variable and often not meaningfully different between groups [11,15]. However, in the included pediatric perioperative and procedural sedation literature, HFNC has not been consistently associated with improved airway procedural success metrics (e.g., first-pass success, number of attempts, or time to definitive airway), and trainee-specific success rates are rarely reported; therefore, the most consistent signal remains oxygenation reliability rather than demonstrable improvement in procedural success.

This distinction matters clinically because pediatric RSI success is frequently determined by whether oxygenation can be maintained continuously through induction and laryngoscopy, especially during common real-world delays (positioning, suctioning, brief interruptions, equipment adjustments), rather than by a uniform ability to tolerate apnea longer in a controlled setting. In other words, HFNC’s most plausible contribution during pediatric airway management may be stabilizing oxygenation during vulnerable windows. This may reduce the frequency or severity of hypoxemia-related interruptions, rather than producing a predictable, uniform extension of apnea tolerance under controlled conditions.

A consistent theme across pediatric airway and physiologic data is that children, particularly infants and younger patients, operate with a smaller oxygen reserve and higher metabolic demand, leading to rapid desaturation during apnea [1,4,8]. Observational registries and multicenter cohorts further show that desaturation events are common and are associated with adverse procedural features (multiple attempts, prolonged attempt duration, esophageal intubation), reinforcing that strategies that improve oxygenation stability may meaningfully affect procedural continuity and safety [3-7]. These findings support using outcome definitions in RSI studies that reflect procedural realities, including interrupted laryngoscopy, rescue ventilation, and severe desaturation, rather than focusing narrowly on surrogate measures such as apnea time alone.

The broader pediatric acute-care HFNC literature strengthens the feasibility argument but must be interpreted cautiously for RSI. In ED and PICU cohorts, HFNC is consistently associated with early improvements in physiologic distress (e.g., heart rate, respiratory rate, oxygenation, work of breathing) and, in many settings, reduced escalation to more invasive support, suggesting it can provide stable oxygenation in high-acuity children [17,21,24]. Meta-analyses in pediatric respiratory distress likewise report reductions in treatment failure and/or intubation compared with standard oxygen and demonstrate non-inferiority versus CPAP in selected populations [21,25]. However, these studies are not RSI-specific and are subject to confounding by indication, variation in escalation thresholds, and differences in comparator oxygen strategies. The more defensible inference is that HFNC is safe and operationally feasible in the age groups and respiratory phenotypes that often require emergent airway management, rather than that it definitively prevents peri-intubation hypoxemia.

Adult peri-intubation evidence provides a useful comparator context but reinforces that the benefit is context-dependent and sensitive to comparator choice. Meta-analyses of ICU intubation and peri-intubation strategies suggest HFNC is generally safe and non-inferior to conventional oxygenation approaches, with improvements in some oxygenation outcomes most apparent in specific subgroups (e.g., less severe baseline hypoxemia or when compared against non-rebreather masks) [18,28]. In emergency surgery, rapid sequence induction, HFNC may improve post-intubation PaO₂ and sometimes prolong apnea time, yet does not consistently reduce desaturation incidence or improve nadir SpO₂ across analyses [19,23,29,30].

Network meta-analyses frequently identify NIV as most protective against hypoxemia in critically ill adults, while still supporting HFNC as superior to standard facemask oxygenation in certain comparisons [28,30]. In children with moderate-to-severe hypoxemia, HFNC may be best viewed as an adjunct within an oxygenation bundle, with a low threshold to escalate to NIV/CPAP when feasible [28,30]. For pediatrics, the key takeaway is not that adult results “prove” HFNC works during RSI, but that study design, including baseline oxygenation severity and what HFNC is compared to, strongly influences apparent effectiveness. This underscores why pediatric RSI trials must be explicit about comparator arms and should not assume HFNC will outperform optimized mask-based preoxygenation or NIV/CPAP strategies in the sickest patients.

From a practical standpoint, current evidence supports considering HFNC as an adjunct within a structured peri-intubation oxygenation bundle, particularly in children with limited reserve (e.g., infants/younger children) and in phenotypes prone to rapid desaturation (e.g., pneumonia, bronchiolitis, mixed respiratory distress), while maintaining readiness to escalate promptly to bag-mask ventilation, supraglottic rescue, or NIV/CPAP when oxygenation deteriorates [15,16,25]. Because HFNC effects are flow- and leak-dependent, protocols should prioritize flow selection that meets or exceeds inspiratory demand, typically using weight-based approaches in smaller children and transitioning to higher fixed flows as size increases [13,16]. However, HFNC should not be positioned as a replacement for disciplined airway preparation, optimized preoxygenation, or a clearly defined rescue plan, particularly given the variability in safe apnea time across pediatric contexts [11,15].

Future pediatric RSI research should prioritize intubation-specific randomized trials with standardized HFNC flow protocols and optimized, clinically relevant comparator arms, including NIV/CPAP strategies for moderate-to-severe hypoxemia when feasible [28,30]. Trials should stratify by age/weight and baseline oxygenation severity, and report key technique factors that influence delivered FiO₂ and distending pressure (e.g., mouth position and leak) [13,16]. Recommended trial endpoints are outlined in the Future Research Priorities and Recommended Trial Endpoints section and should emphasize oxygenation reliability and procedural continuity rather than apnea time alone [4,6,11].

Limitations

This review should be interpreted in light of several important limitations inherent to the available evidence base and to the narrative “best-evidence” approach. First, direct evidence evaluating HFNC specifically during pediatric RSI is sparse, and much of the pediatric literature informing peri-intubation oxygenation comes from adjacent contexts (e.g., procedural sedation, anesthesia induction, bronchiolitis/respiratory distress cohorts, and neonatal practice). As a result, several inferences about peri-intubation performance rely on indirectness rather than RSI-specific randomized comparisons. Second, the included studies are heterogeneous in patient age and acuity, setting (ED, PICU, OR), clinical indication, and oxygenation targets, with substantial variability in HFNC protocols (e.g., flow selection, timing as preoxygenation vs apneic oxygenation, FiO₂ settings, interface sizing) and in comparator strategies (e.g., standard nasal cannula, facemask/non-rebreather, bag-mask ventilation, NIV/CPAP). This heterogeneity limits cross-study comparability and precludes precise effect estimation in a qualitative synthesis.

Third, outcomes and definitions are inconsistently reported across studies; clinically meaningful endpoints for peri-intubation performance (e.g., severe desaturation thresholds, time below saturation cutoffs, interrupted laryngoscopy, rescue ventilation, first-pass success, and adverse events) are not uniformly measured, and “safe apnea time” is often defined differently or not measured in a standardized fashion. Fourth, observational pediatric data are vulnerable to confounding by indication and practice-pattern effects (e.g., operator experience, airway difficulty, preoxygenation quality, use of positioning, suctioning, and rescue thresholds), which may influence both HFNC selection and outcomes. Fifth, adult peri-intubation trials and network meta-analyses were included to provide comparator context and to illustrate how baseline hypoxemia severity and comparator choice can shape apparent effectiveness; however, adult physiology and clinical context differ materially from pediatrics, limiting direct generalizability.

Finally, because this is a narrative best-evidence review rather than a PRISMA-guided systematic review, we did not perform duplicate screening, quantitative pooling, or a formal risk-of-bias synthesis across all included studies, and publication bias cannot be excluded. Collectively, these limitations underscore the need for pediatric RSI-specific randomized trials with standardized flow protocols, optimized comparator arms, stratification by baseline hypoxemia severity and age/weight, and consistent reporting of clinically meaningful peri-intubation outcomes.

## Conclusions

HFNC is a physiologically plausible and operationally feasible adjunct for pediatric RSI, but the most defensible interpretation of the current literature is that its benefit, when present, is more consistently associated with improved oxygenation reliability rather than a predictable prolongation of safe apnea time. Across pediatric perioperative and sedation studies and broader ED and PICU experience, HFNC is most often associated with fewer desaturation events, higher minimum SpO₂, and fewer rescue interventions, while effects on apnea duration and CO₂ endpoints remain inconsistent. Adult peri-intubation trials provide useful context, emphasizing that effectiveness depends heavily on baseline hypoxemia and the comparator strategy, with NIV frequently preferred in cases of more severe hypoxemia, underscoring the importance of cautious extrapolation to pediatric practice. Overall, HFNC can be considered a component of a structured peri-intubation oxygenation bundle, particularly for younger children and those with limited reserve, while maintaining readiness to escalate to mask ventilation, supraglottic rescue, or NIV or CPAP. Definitive guidance now requires pediatric RSI-specific randomized trials with standardized flow protocols, explicit comparators, and endpoints that reflect real-world airway performance and clinically meaningful hypoxemia.
